# Exercise Performance and Corticospinal Excitability during Action Observation

**DOI:** 10.3389/fnhum.2016.00106

**Published:** 2016-03-16

**Authors:** James G. Wrightson, Rosie Twomey, Nicholas J. Smeeton

**Affiliations:** ^1^Welkin Human Performance Laboratory, Centre for Sport and Exercise Science and Medicine, University of BrightonEastbourne, UK; ^2^Department of Sport, Exercise and Rehabilitation, Faculty of Health and Life Sciences, Northumbria UniversityNewcastle upon Tyne, UK

**Keywords:** motor resonance, TMS, deception, mirror neurons, sport performance, action observation network

## Abstract

**Purpose:** Observation of a model performing fast exercise improves simultaneous exercise performance; however, the precise mechanism underpinning this effect is unknown. The aim of the present study was to investigate whether the speed of the observed exercise influenced both upper body exercise performance and the activation of a cortical action observation network (AON).

**Method**: In Experiment 1, 10 participants completed a 5 km time trial on an arm-crank ergometer whilst observing a blank screen (no-video) and a model performing exercise at both a typical (i.e., individual mean cadence during baseline time trial) and 15% faster than typical speed. In Experiment 2, 11 participants performed arm crank exercise whilst observing exercise at typical speed, 15% slower and 15% faster than typical speed. In Experiment 3, 11 participants observed the typical, slow and fast exercise, and a no-video, whilst corticospinal excitability was assessed using transcranial magnetic stimulation.

**Results**: In Experiment 1, performance time decreased and mean power increased, during observation of the fast exercise compared to the no-video condition. In Experiment 2, cadence and power increased during observation of the fast exercise compared to the typical speed exercise but there was no effect of observation of slow exercise on exercise behavior. In Experiment 3, observation of exercise increased corticospinal excitability; however, there was no difference between the exercise speeds.

**Conclusion**: Observation of fast exercise improves simultaneous upper-body exercise performance. However, because there was no effect of exercise speed on corticospinal excitability, these results suggest that these improvements are not solely due to changes in the activity of the AON.

## Introduction

An athlete rarely performs in isolation. The track cyclist, the 1500 m runner and the wheelchair marathoner will often be surrounded by, and observe, other athletes when performing. For over a century it has been recognized that the presence of a competitor influences an athlete’s performance (Triplett, 1898; Wilmore, 1968). Proposed mechanisms for these improvements have predominantly focused on arousal, motivation and affect (Brehm and Self, 1989; Renfree et al., 2014). Recently, a number of groups have reported improved exercise performance when the speed of the observed model was deceptively increased (Corbett et al., 2012; Stone et al., 2012; Williams et al., 2015). These findings suggest that the effect of competition on exercise performance may be influenced by the speed of the competitor. However, the neuroscientific mechanisms of this effect remain un-investigated. Understanding why the speed of the observed exercise mediates exercise performance may allow practitioners to enhance training and competition by providing relevant visual stimuli.

Psychophysiological explanations for the effect of the observed action on simultaneous exercise performance state that competition against the model increases motivational and arousal states, allowing access to a centrally controlled anaerobic physiological reserve (Corbett et al., 2012) in line with suggested models of the central regulation of exercise performance (Noakes, 2012). Evidence for this suggestion comes from studies in which participants competed against an avatar whose speed through the virtual environment was deceptively increased above the participants’ previous best performance (Corbett et al., 2012; Stone et al., 2012; Williams et al., 2015). A possible confounding factor in these studies; however, is the change in optic flow during the fast videos. Optic flow has been shown to influence perception of effort during cycling (Parry et al., 2012) which may influence pacing strategies and thus alter exercise performance (Tucker, 2009). Alternatively, Eaves et al. (2008) suggested that changes in exercise performance during action observation may instead be caused by incidental visual coupling between observer and model, which leads to an altered limb recruitment strategy and therefore a more efficient limb co-ordination pattern and improved exercise efficiency.

Another possible mechanism for the effect of action observation on exercise performance is that the exercise speed observed modifies the activation of a cortical action observation network (AON). Action observation has been suggested to activate neural circuits involved in action execution. This is termed motor resonance, whereby the mapped visual representation facilitates subsequent execution of the observed action (Jeannerod, 2001; Vogt and Thomaschke, 2007; Eaves et al., 2012, 2014). A number of studies have used Transcranial Magnetic Stimulation (TMS) to examine motor resonance in discrete reaching and grabbing actions (e.g., Aglioti et al., 2008; Loporto et al., 2013) and reported that action observation increased corticospinal excitability, suggested to represent increased activity in the AON (Fadiga et al., 2005), and force production (Porro et al., 2007). Borroni et al. (2005) reported that corticospinal excitability in the extensor carpi radialis (ECR) during action observation of cyclical wrist extension and flexion was dependent on the phase of movement (and thus observed muscle activation) suggesting that kinematic movement characteristics, such as movement speed, may mediate AON activity. Therefore, speed dependent changes in AON activity may underlie performance changes during observation of fast exercise. However, although action observation may be a promising ergogenic tool for sport training (Holmes and Calmels, 2008), in contrast to the well characterized effects of observation of a discrete action on corticospinal excitability (Vogt and Thomaschke, 2007), very little is known about the effects of the cyclical actions typical of many sports. Manipulating the speed of an observed exercise task may provide a novel training stimulus. However, the mechanisms underpinning changes in exercise performance in response to manipulation of the observed action speed remain unclear. Therefore, the present study had two aims, which we addressed over three experiments:

The first aim was to examine the effect of observation of a fast exercise on upper-body arm-crank exercise performance (Experiments 1 and 2). We predicted that observation of a fast exercise would improve exercise performance, replicating effects seen in lower-body exercise (Corbett et al., 2012; Stone et al., 2012). However, in contrast to these previous studies, we used a stationary model for observation to control for the possibly confounding influence of changes in optic flow on exercise performance (Parry et al., 2012). The second aim was to examine the effect of the speed of an observed exercise on activation of a cortical AON measured using TMS (Experiment 3). Because observation may influence exercise behavior through incidental coupling (Eaves et al., 2008) we predicted that observation of a slow, fast, and typical video would result in video speed dependent changes in cadence. Changes in MEP amplitude coinciding with changes in video speed would implicate the AON in this coupling.

## Experiment 1

### Materials and Method

#### Participants

Ten (one female) recreationally active participants (mean ± SD age = 19.5 ± 0.5 years) were recruited for Experiment 1. The procedures of Experiments 1–3 received ethical approval from the University ethics committee. Prior to participation and following a full explanation of procedures, participants provided written informed consent for individual experiments.

#### Stimuli and Apparatus

In all three experiments, all arm crank exercise trials were performed on an upright adapted cycle ergometer (SRM, Jülich, Germany) with an inbuilt power meter. Strain gages and a reed contact switch in the crank sampling at 2 Hz recorded average torque and cadence (rev.min^-1^) to give power (W). The ergometer was in the “open ended” mode, where resistance is provided by the electromagnetic brake based on the angular velocity and torque at the crank. Analysis of the ergometer data was performed oﬄine (SRM training system version 6, SRM, Jülich, Germany).

The observational stimuli were recorded using a digital video camera (HC-V100, Panasonic, Osaka, Japan) positioned behind a model performing arm-crank exercise for 3 min. All videos were edited oﬄine using a video editing package (Premiere 5, Adobe, San Jose, CA, USA). The 3 min clip was then looped so each video was 18 min in length. Subsequently, two separate videos were created for each participant in which the speed of the video was manipulated so that the model’s cadence matched either the participant’s mean cadence during a familiarization time trial (typical speed video; TYP) or 15% above their mean time trial cadence (TYP+15) (see below). The observation videos were projected from a personal computer onto a 125 cm × 145 cm screen placed 200 cm in front of the arm-crank ergometer, via a digital projector with a resolution of 800 × 600 pixels which created a visual angle subtending to 38° (H) and 40° (W).

Breath by breath oxygen uptake ($\overset{\cdot }{V}$O_2_) was recorded using an automated online metabolic cart (Metalyser sport, Cortex, Lepzig, Germany) which was calibrated before each trial using standardized gas samples (15.10% O_2_, 5.06% CO_2_) and a 3 l syringe. Ratings of perceived exertion (RPE) were determined using the Borg scale (Borg, 1970) and heart rate (b.min^-1^) was recorded using a wireless telemetry system (Polar electro, Kimpele, Finland).

#### Procedure

During an initial visit to the laboratory, participants performed a 5 km time trial. Participants were given onscreen feedback regarding speed (m.s^-1^), distance covered (m) and time (s). Participants were instructed to reach 5 km as quickly as possible. Each participant’s mean cadence over the time trial was recorded and used to create their individualized observation videos.

Following this initial visit and video creation, participants attended the laboratory on three separate occasions, separated by at least 48 h. In each of these action observation trials, and after a 3 min warm up, participants performed a 5 km time trial whilst observing either a fixation point on the blank screen (no-video), observing the TYP video, or the TYP+15. Video order was randomized across trials and participants were naive to the speed manipulation. Participants received feedback on the distance covered every kilometer. Every 500 m, participants were given standardized verbal encouragement. In all experiments participants were monitored throughout to ensure they were watching the action on the screen and given verbal prompts to “keep looking at the screen” or to “keep watching the action on the screen”. Participants were notified when there was 500 m remaining. Power (W), cadence (rev.min^-1^) and VO_2_ (ml.kg^-1^.min^-1^) were recorded throughout trials. Every kilometer, heart rate (b.min^-1^) and RPE (Borg, 1970) were recorded. Time taken (s) to complete 5 km (performance time) was recorded at the end of each trial.

#### Data Analysis

All data are presented as mean [95% CI]. All data were assessed for normality using the Kolmogorov–Smirnov test. The effect of the speed of the observed exercise (video speed) on performance time and mean power, cadence, VO_2_, heart rate and RPE was examined using a one way repeated measure ANOVA with Video Speed (no-video, TYP and TYP+15) as the independent variable. Significant effects were followed up using pre-planned contrasts (no-video vs. TYP, no-video vs. TYP+15). For all experiments, statistical significance was set at *p* < 0.05. Partial eta squared ($\eta_{p}^{2}$) was used as measure of effect size.

### Results

**FIGURE 1:**
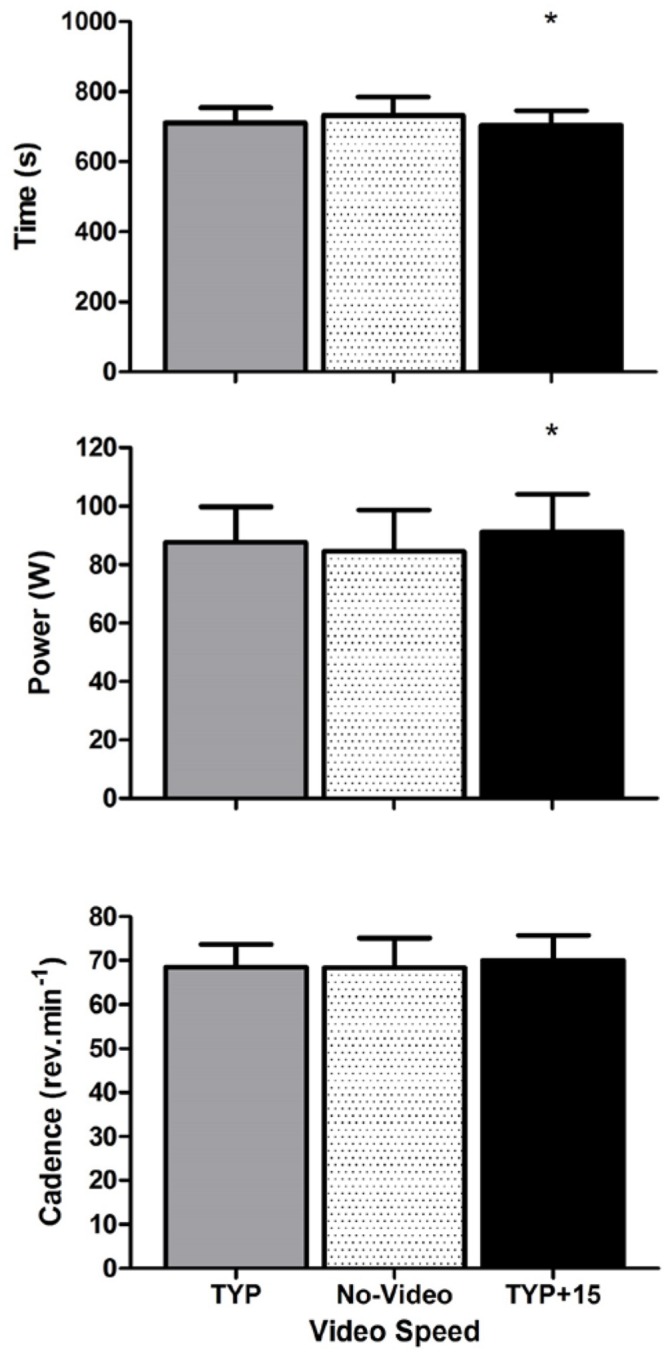
**Experiment 1, mean [95% CI] performance time (s), power (W), and cadence (rev.min^-1^) at each video speed**. ^∗^denotes a significant difference between video speeds. TYP, typical speed video; TYP+15, video speed = 15% faster than typical speed.

Mean [95% confidence intervals] for all dependent variables across all three experiments are displayed in **Table 1**. Mean cadence during the familiarization 5 km was 70 [66–74] rpm and mean performance time was 732 [677–787] s. **Figure 1** shows the effect of video speed on performance time, power, and cadence. ANOVA revealed an effect of Video Speed on performance time [*F*_(2,18)_= 6.5, *p* = 0.008, $\eta_{p}^{2}$ = 0.42]. Pre-planned contrast revealed that participants completed the 5 km faster during TYP+15 than during no-video [*F*_(1,9)_= 10.5, *p* = 0.010, $\eta_{p}^{2}$ = 0.54]. There was no difference between the TYP and no-video conditions [*F*_(1,9)_= 4.2, *p* = 0.071, $\eta_{p}^{2}$ = 0.32].

**Table 1:** Mean [95% CI] for all dependent variables across all three experiments.

		Video speed			
Experiment	Variable	No-video	TYP	TYP+15	TYP–15
1	Time (s)	732.0 [679.2-784.8]	711.5 [668.6-754.4]	703.5 [661.2-745.8]	
	Power (W)	84.6 [70.5-98.7]	87.6 [75.5-99.7]	91.1 [78.0-104.1]	
	Cadence (rev.min^-1^)	68.6 [63.5-73.7]	68.4 [61.7-75.1]	70.1 [64.3-75.8]	
	RPE (Borg)	13.4 [12.4-14.3]	13.7 [13.0-14.3]	14.3 [13.3-15.3]	
	Heart Rate (b.min^-1^)	146.4 [131.9-160.9]	149.3 [141.2-157.3]	154.7 [139.6-169.7]	
2	Power (W)		67.5 [60.6-74.4]	69.8 [63.1-76.5]	66.7 [60.1-73.3]
	Cadence (rev.min^-1^)		76.0 [58.5-93.4]	81.9 [62.6-101.1]	74.9 [58.7-91.1]
3	MEP amplitude (mV)	0.43 [0.27-0.58]	0.51 [0.36-0.66]	0.50 [0.33-0.66]	0.54 [0.35-0.73]

*TYP, typical speed video; TYP+15, video speed = 15% faster than typical speed; TYP–15, video speed = 15% slower than typical speed.*

There was also an effect of Video Speed on power [*F*_(2,18)_= 3.661, *p* = 0.046, $\eta_{p}^{2}$ = 0.29]. Mean power was higher during TYP+15 than during the no-video condition [*F*_(1,9)_= 8.9, *p* = 0.015, $\eta_{p}^{2}$ = 0.50]. There was no difference in total power between TYP and the no-video condition [*F*_(1,9)_= 1.7, *p* = 0.231, $\eta_{p}^{2}$ = 0.155]. There was no effect of Video Speed on cadence [*F*_(2,18)_= 0.8, *p* = 0.431, $\eta_{p}^{2}$ = 0.09].

There was an effect of Video Speed on RPE [*F*_(2,18)_= 3.649, *p* = 0.047, $\eta_{p}^{2}$ = 0.29] where RPE was significantly higher [*F*_(1,9)_= 6.4, *p* = 0.032, $\eta_{p}^{2}$ = 0.42] during TYP+15 (14.3) than the no video condition (13.4) but there was no difference between TYP (13.7) and no-video [*F*_(1,9)_= 2.1, *p* = 0.177, $\eta_{p}^{2}$ = 0.19]. Due to equipment malfunction, heart rate was not recorded for one participant and $\overset{\cdot }{V}$O_2_ was not recorded for two participants. There was no effect of video speed on heart rate [*n* = 9, *F*_(2,16)_= 1.6, *p* = 0.228, $\eta_{p}^{2}$ = 0.17, pooled mean = 150+16 b.min^-1^] or $\overset{\cdot }{V}$O_2_ [*n* = 8, *F*_(14,2)_= 1.6, *p* = 0.855, $\eta_{p}^{2}$ = 0.02, pooled mean = 1.97+ 0.36 ml.kg^-1^.min^-1^].

## Experiments 2 and 3

The main finding of Experiment 1 was that observation of a fast exercise improved arm-crank time trial performance compared to observation of the no video condition, whilst observation of typical speed exercise did not influence performance replicating previously reported effects in lower body exercise (Corbett et al., 2012; Stone et al., 2012). This result suggests the observation effect on performance cannot only be attributed to optical flow. However, because observation of different limb actions (Eaves et al., 2008) and AON activity (Borroni et al., 2005) may also explain the effect seen on exercise behavior, speed of exercise observed was also made slower as well as faster to directly examine the effect limb speeds observed on the exercise behavior and AON activity. To replicate the effect of Experiment 1, it was expected that measures of exercise performance would be higher in the fast video speed condition (TYP+15) compared to typical (TYP). Furthermore, to test the direct effect, exercise performance was also expected to be lower in the slow video speed condition (TYP-15) compared to typical (TYP) (Experiment 2). In addition, if AON mediated the observation effect on exercise performance in Experiment 1, then corticospinal excitability would be different in the TYP-15 and Typ+15 conditions compared to the TYP condition (Experiment 3).

### Materials and Method

#### Participants

Eleven (four female) recreationally active healthy young adults (mean±SD age = 22.6 ± 2.3 years) took part in Experiment 2 and 11 (five female) recreationally active healthy young adults (mean + SD age = 21.1 ± 2.2 years) were recruited into Experiment 3. In Experiment 3, handedness was assessed using the Edinburgh handedness scale (Oldfield, 1971). One participant was left handed.

#### Stimuli and Apparatus

The stimuli consisted of two 9 min videos, each comprising three, 3 min clips. The speed of each 3 min clip was manipulated so that the cadence of the model matched either the participant’s typical cadence (TYP), or a cadence 15% above (TYP+15) or 15% below (TYP–15) their typical cadence recorded during a baseline time trial (described below). Each 9 min video was thus comprised of 3 min of each of TYP, TYP+15, and TYP–15. Two videos were created for each participant and the order of each clip was counter balanced across trials and participants to control for order effects. Participants were naive to this manipulation, and were told that the aim of the experiment was to examine the effect of action observation on arm-crank performance.

In Experiment 3, corticospinal excitability was assed using a figure of eight coil (70 mm diameter) powered by a mono-pulse magnetic stimulator (Magstim 200^2^, The Magstim company, Whitland, UK). The coil was placed over primary motor cortex contralateral to the dominant hand (right hemisphere for the left handed participant) using the 10–20 system (Herwig et al., 2003) with the coil tangential to the scalp and at 45° to the midline so that the current flowed in an anterior-posterior direction. The coil was placed over the point at which single pulse stimulation delivered at 50% of maximum stimulator output elicited the largest motor evoked potential (MEP) in the right ECR and this was marked on the scalp with an indelible pen. Evoked responses were recorded using surface electromyography. Single use electrodes (H59P, Kendall, Mansfield, MA, USA) were placed 1 cm apart on the belly of the ECR (defined as 1/3 of the distance between the lateral epicondyle and the styloid process of the radius). Signals were amplified (x1000), band-pass filtered (20–2000 Hz), digitized (4 kHz), recorded, and analyzed oﬄine in the LabChart 7 software via a Powerlab 26T digital to analog interface (ADInstruments, Oxford, UK). The stimulator output equal to motor threshold was determined using an adaptive estimation method (Awiszus, 2003, 2011). The TMS motor threshold assessment tool (MTAT v2.0, Awiszus and Borckardt, 2010) was used to run the maximum-likelihood threshold-tracking algorithm. All subsequent stimulations were delivered at 120% of motor threshold. The mean peak-to-peak amplitude of the evoked MEPs was used as marker of corticospinal excitability/motor resonance (Fadiga et al., 1995).

### Procedure

Initially, participants performed a 10 km arm-crank time trial with no observation stimuli. The mean cadence between kilometers 3–8 was recorded as the typical cadence for the time trial.

In Experiment 2 participants were asked to perform two 9 min arm-crank exercise bouts whilst observing the observation videos. Cadence (rev.min^-1^) and power (W) were recorded by the ergometer during each 9 min exercise bout. After completing the exercise bout, participants rested for 10 min before the procedure was repeated with the second observation video. The data for each 9 min exercise bout was analyzed oﬄine using the SRM training system (version 6, SRM, Jülich, Germany).

In Experiment 3, participants were asked to observe a blank screen (no-video) during which they received 25 stimulations (Bastani and Jaberzadeh, 2012) delivered with an inter-stimulus gap of >3 s. Participants were instructed keep their arm relaxed. Background EMG was visually inspected prior each stimulation to ensure there was no muscle activity. Subsequently, each participant was shown two 9 min videos each comprised of the three video clips (TYP, TYP+15, and TYP–15) whilst again receiving 25 stimulations.

#### Data Analysis

Initially, differences in power, cadence, and MEP amplitude between the two 9-min videos were examined using a two tailed Student’s *t*-test. As no statistically significant differences were found (all *p* > 0.05), data was collapsed across both bouts. The effect of the speed of the observed exercise was examined using one way repeated measures ANOVAs with the independent variable observation video speed having three levels (TYP, TYP+15 and TYP–15) for exercise performance and four levels (no-video, TYP, TYP+15, and TYP–15) for corticospinal excitability. Significant effects were followed up using pre-planned contrasts: [TYP vs. TYP+15] and (TYP vs. TYP–150) for exercise performance and (no-video vs. all video conditions), (TYP vs. TYP+15 and TYP–15), then (TYP+15 vs. TYP–15) for corticospinal excitability. Statistical significance was set at *p* < 0.05 and partial eta squared ($\eta_{p}^{2}$) values were used to measure effect sizes.

### Results

#### Exercise Behavior

**FIGURE 2:**
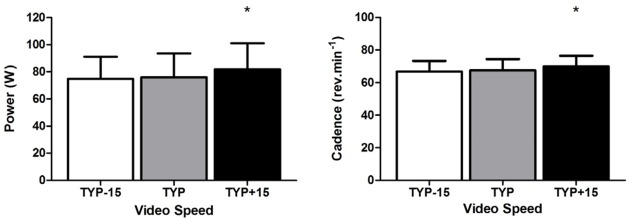
**Experiment 2, mean [95% CI] power (W) and cadence (rev.min^-1^) across all three video conditions**. ^∗^denotes a significant difference between TYP+15 and TYP video conditions. TYP, typical speed video; TYP–15, video speed = 15% slower than typical speed; TYP+15, video speed = 15% faster than typical speed.

There was a significant effect of video speed on mean power [*F*_(2,20)_ = 5.64, *p* = 0.011, $\eta_{p}^{2}$ = 0.36]. Power was higher during TYP+15 than during TYP [*F*_(1,10)_ = 7.810, *p* = 0.019, $\eta_{p}^{2}$ = 0.44], there was no difference in power between TYP and TYP-15 [*F*_(1,10)_ = 0.27, *p* = 0.62, $\eta_{p}^{2}$ = 0.03, **Figure 2**].

Mean typical cadence during the 3rd to the 8th km during the 10 km time trial was 62 [56–68] rev.min^-1^. Repeated measures ANOVA revealed a significant effect of Video Speed on mean cadence [F_(2,20)_ = 9.65, *p* = 0.001, $\eta_{p}^{2}$ = 0.49]. Pre-planned contrasts revealed cadence was higher during TYP+15 than during TYP [*F*_(1,10)_ = 16.0, *p* = 0.003, $\eta_{p}^{2}$ = 0.62, **Figure 2**]. There was no difference in cadence between TYP and TYP-15 [*F*_(1,10)_ = 0.84, *p* = 0.38, $\eta_{p}^{2}$ = 0.08].

#### Corticospinal Excitability

**FIGURE 3:**
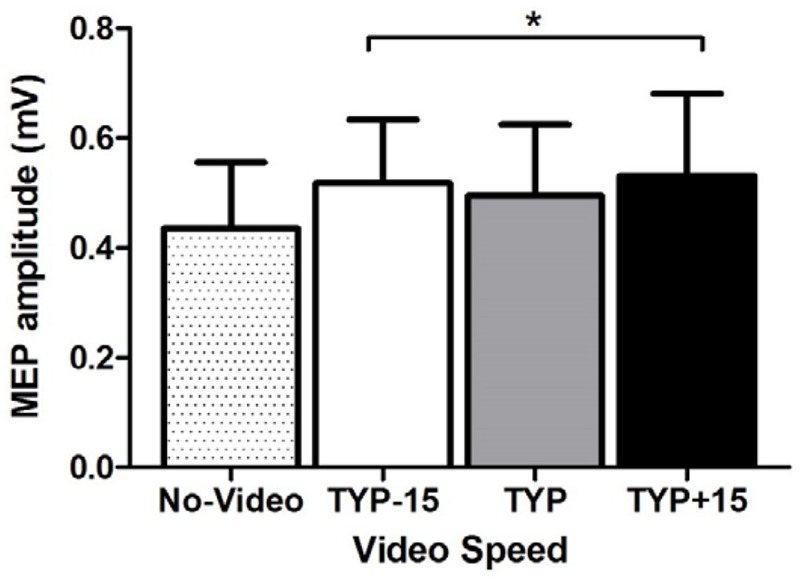
**Experiment 3, mean [95% CI] power (W) and cadence (rev.min^-1^) across all three video conditions**. ^∗^denotes a significant difference between no-video and all video conditions. TYP, typical speed video; TYP–15, video speed = 15% slower than typical speed; TYP+15, video speed = 15% faster than typical speed.

ANOVA revealed an effect of video speed on MEP amplitude [*F*_(3,30)_ = 3.2, *p* = 0.037, $\eta_{p}^{2}$ = 0.24, **Figure 3**]. Pre-planned contrasts revealed a significant difference between the no-video condition and all video conditions [*F*_(1,10)_ = 6.7, *p* = 0.027, $\eta_{p}^{2}$ = 0.40] where MEP amplitude was higher during the video conditions than during the no-video condition (**Figure 3**). There was no difference between TYP and the two manipulated video speeds [*F*_(1,10)_ < 0.1, *p* = 0.882, $\eta_{p}^{2}$ < 0.01]. There was also no difference between TYP+15 and TYP-15 [*F*_(1,10)_ = 1.8, *p* = 0.207, $\eta_{p}^{2}$ = 0.15].

## Discussion

The present study examined the effect of the speed of an observed cyclical exercise on exercise performance and activation of the AON. As hypothesized, observation of fast exercise improved upper-body cyclical exercise performance, extending the findings of previous studies in lower body cycling (Corbett et al., 2012; Stone et al., 2012). However, whilst observation of fast exercise improved exercise performance there was no effect of observation of slow exercise on exercise performance. In addition, although observation of cyclical exercise increased corticospinal excitability, there was no effect of speed of the observed exercise. Taken together, these findings support the suggestion that improvements to exercise performance may be due to changes in arousal and affect (Jones et al., 2013), rather than changes to the activity of the AON.

In Experiment 1, we examined whether observation of fast and typical speed upper-body exercise improved simultaneous exercise performance. The main finding was that observation of a fast but not typical speed exercise improved performance in a 5 km arm-crank time trial. The improvement in time trial performance occurred without changes to oxygen uptake. These findings are in accordance with recent studies that have shown that the speed of an observed action influences lower-body cyclical exercise performance through an increased use of anaerobic energy systems when observing, and competing against, a model (Corbett et al., 2012; Stone et al., 2012). Although we did not explicitly test the contribution of each energy system during action observation, the results of Experiment 1 extend these findings to show that observation of a fast action also improves upper-body cyclical exercise performance with little or no changes to oxygen utilization during performance. In addition, unlike in previous studies (Corbett et al., 2012; Stone et al., 2012) we controlled for the possibly confounding effect of optical flow on exercise performance (Parry et al., 2012), by having the model exercise model on a stationary ergometer. These results thus indicate that performance improvements during observation of fast exercise are not solely due to changes in optical flow.

In Experiment 2, we examined the effect of observation of both slow and fast exercise on exercise performance. Participants increased their cadence and power during observation of fast exercise, however, observation of slow exercise did not change exercise performance. Observation of both fast and slow discrete actions mediates the speed of a subsequently executed action (Bove et al., 2009) and Eaves et al. (2008) reported incidental visual coupling between the observer and model led to alterations in the observers’ limb co-ordination and movement efficiency. In contrast, here we found that the effect of exercise speed during observation of cyclical exercise occur only during observation of fast exercise. One possible interpretation of these findings is that the effects of observation of fast exercise on exercise performance are not due to a general mechanism of visual coupling between observer and model, because the cadence of the observer was not altered during observation of slow exercise, only during observation of fast exercise. However, it is possible that mechanical properties of the neuromuscular system prevent the adoption of a slow cadence. Whilst the mechanical and neurophysiological mechanisms underpinning freely chosen cadence in cyclical exercise are complex and not fully understood, both elite (Foss and Hallén, 2005) and less experienced cyclists (Marsh and Martin, 1997) choose a cadence higher than their most mechanically efficient. Proposed mechanisms for the selection of high cadences include the percentage of muscle fiber type in the active muscles (Hansen et al., 2002) and the force–velocity relationship of the active muscle fibers (Kohler and Boutellier, 2005), or the control of temporal agonist and antagonist muscle activity by innate central pattern generators. Regardless of the mechanisms, it is possible that adoption of a slow movement pattern during observation of slow exercise may be prevented by mechanical and neurophysiological properties of the motor system. In contrast, observation of a fast exercise may interact with some of the mechanisms which underpin the adoption of higher cadences, such as alterations in the top–down influences on CPG activity.

In Experiment 3, we examined the effect of observation of both fast and slow exercise on corticospinal excitability. The main finding was that observation of cyclical upper-body exercise increased corticospinal excitability. This is the first time that observation of cyclical exercise have been shown to increase corticospinal excitability, extending previous findings in discrete actions (Aglioti et al., 2008; Loporto et al., 2013). Increases in corticospinal excitability during action observation are suggested to represent “motor resonance” in the AON, mapping visual representation to motor knowledge to allow understanding of the action (Jeannerod, 2001; Rizzolatti and Craighero, 2004; Fadiga et al., 2005). Activation of neural pathways in response to action observation may improve motor performance and learning by priming neural structures used during action execution and producing training adaptations to the central nervous system without increasing training loads, potentially reducing training related fatigue (Holmes and Calmels, 2008). However, the AON effect observed here was not mediated by the speed of the observed exercise. Agosta et al. (2016) recently reported that corticospinal excitability during observation of upper limb motion was dependent on movement kinematics and the velocity profile of the observed action. Previously, Borroni et al. (2005) reported that corticospinal excitability during action observation of wrist extension and flexion was dependent on the phase of movement (and thus observed muscle activation), suggesting that changes to movement kinematics, such as action speed, may mediate the effects of action observation. However, the control of discrete and cyclical actions may be reliant on different processes (Huys et al., 2008). Indeed, Takahashi et al. (2008) reported an increase in corticospinal excitability during observation of gait which was independent of observed muscle activation. It is therefore possible that velocity dependent changes to corticospinal excitability during action observation are themselves dependent on characteristics of the observed task. Additionally, while the present results may indicate that the speed of the observed action does not influence AON activity; temporal brain activity has been reported to change during action observation (Cochin et al., 1998; Rizzolatti et al., 2001; Pineda, 2005). It is possible that there may be temporal entrainment between observed action kinematics and AON activity which cannot be examined using TMS. Further research is thus warranted to examine the effect of the speed of an observed cyclical action on temporal cortical activity. Manipulation of the speed of an observed exercise could thus present a novel way to elicit neuro-plastic changes to the central nervous system to complement existing action observation training methods.

## Conclusion

These three experiments have shown for the first time that observation of fast exercise improves simultaneous upper-body exercise performance, extending the previous data reporting the same effect in cycling (Corbett et al., 2012; Stone et al., 2012). These results support the proposal that this effect is at least in part due to increases in motivation and arousal as observers compete against the model (Corbett et al., 2012) and not solely due to changes in optical flow. In addition, simultaneous exercise performance during action observation of cyclical actions may be constrained by innate properties of the motor system that influence cadence selection. We have also shown, for the first time, that observation of cyclical exercise increases corticospinal excitability and is not dependent on exercise speed. A fuller understanding of the effects of action observation on visual and neural systems is required before its suitability as an adjunctive training tool can be assessed.

## Author Contributions

JW, RT, and NS all made substantial contributions to the conception and design of the study. JW and RT were responsible for data acquisition and analysis. JW, RT, and NS all made substantial contributions to the interpretation of the data drafting revision and final approval of the article.

## Conflict of Interest Statement

The authors declare that the research was conducted in the absence of any commercial or financial relationships that could be construed as a potential conflict of interest.
